# Evaluation of pH in Sausages Stuffed in a Modified Casing with Orange Extracts by Hyperspectral Imaging Coupled with Response Surface Methodology

**DOI:** 10.3390/foods11182797

**Published:** 2022-09-10

**Authors:** Chao-Hui Feng, Hirofumi Arai, Francisco J. Rodríguez-Pulido

**Affiliations:** 1 School of Regional Innovation and Social Design Engineering, Faculty of Engineering, Kitami Institute of Technology, 165 Koen-cho, Kitami, Sendai 090-8507, Japan; 2 RIKEN Centre for Advanced Photonics, RIKEN, 519-1399 Aramaki-Aoba, Aoba-ku, Sendai 980-0845, Japan; 3 Food Colour and Quality Laboratory, Facultad de Farmacia, Universidad de Sevilla, 41012 Sevilla, Spain

**Keywords:** casing modification, orange extracts, hyperspectral imaging, response surface methodology, partial least squares regression

## Abstract

The pH values of sausages stuffed in natural hog casings with different modifications (soy lecithin, soy oil, orange extracts (OE) from waste orange peels, lactic acid in slush salt, and treatment time) after 16-day 4 °C storage were evaluated for the first time by hyperspectral imaging (350–1100 nm) coupled with response surface methodology (RSM). A partial least squares regression (PLSR) model was developed to relate the spectra to the pH of sausages. Spectral pretreatment, including first derivative, second derivative, multiplicative scatter correction (MSC), standard normal variate (SNV), normalization, and normalization, with different combinations was employed to improve model performance. RSM showed that only soy lecithin and OE interactively affected the pH of sausages (*p <* 0.05). The pH value decreased when the casing was treated with a higher concentration of soy lecithin with 0.26% OE. As the first and second derivatives are commonly used to eliminate the baseline shift, the PLSR model derived from absorbance pretreated by the first derivative in the full wavelengths showed a calibration coefficient of determination (R^2^) of 0.73 with a root mean square error of calibration of 0.4283. Twelve feature wavelengths were selected with a comparable R^2^ value compared with the full wavelengths. The prediction map enables the visualization of the pH evolution of sausages stuffed in the modified casings added with OE.

## 1. Introduction

Due to their special bite and unique flavor, natural casings are widely utilized in sausage production. Nevertheless, the high bursting incidence hinders the rapid and efficient large-scale manufacture of sausages. Nature casings are required to bear the high pressures generated during stuffing or precooking without compromising sausage quality. Previous studies have proven that casings can become more porous after modification and reduce the bursting that occurs during immersion vacuum cooling [1]. Due to the change in the microstructure of modified casings, microorganism invasion and oxidation can easily occur; therefore, a food-grade antioxidative or antimicrobial reagent is required to prolong the shelf life of modified casings for better commercial utilization.

Citrus fruit peels, which possess a large amount of useful and helpful biologically active compounds for human beings, are currently either directly discarded to landfills as fertilizer, used as animal feed, or sold as dried orange peels in China, serving as one of the ingredients of Chinese herbal medicine [2]. The waste of orange peels not only causes environmental problems, but it is also highly costly to deal with waste in accordance with the Food Recycling Law in Japan [3]. As a member of the flavonoids family and rich in the peels of citrus fruits, hesperidin has been demonstrated to be effective against liver cancer and lung cancer [4], possessing antioxidant [5] and antimicrobial properties [6,7]. The latest literature also reveals that flavonoids such as hesperidin and rutin can be regarded as the starting point for therapeutics against COVID-19 [8]. If the waste of orange peels can be taken full advantage of, it can not only reduce environmental issues but also utilize value-added products and increase industrial income [9]. As modified casings require an antioxidative or antimicrobial reagent, it is meaningful to add extracts from these waste orange peels. Fernández-López (2007) studied the quality of dry-cured sausages according to different concentrations of orange fiber powder (mixture of albedo, flavedo, and pulp) with a 28-day dry-curing time of 15 ± 1 °C and relative humidity of 75% [10]. The pH values of all samples decreased during the first week and then returned to similar values after 4 weeks. Fernández-Ginés et al. (2003) observed that a lower pH value was obtained when a higher concentration of orange fiber was used [11]. The reason for using citrus fiber was that it possesses better quality than other dietary fibers due to the presence of bioactive compounds such as flavonoids, polyphenols, carotenes, and antioxidant properties [11].

Response surface methodology (RSM) is a useful statistical method that can interpret the relationship between food processing parameters and the quality attributes of food products [12,13,14,15,16,17], especially if the effects of variables on the response are unknown. As a powerful design that can greatly reduce the number of experiments compared with a full experimental design, the central composite design (CCD) has been employed in several studies by RSM with a single run of different variable combinations and replicates only at the center point [18,19,20]. Currently, however, there are no relevant studies on combining sausage casing modification and orange extracts from waste orange peels by using RSM, which merits investigating their effects on sausage quality.

Hyperspectral imaging (HSI) is more advantageous than conventional spectroscopic methods, as it can provide the spectral information and spatial distribution of a subject simultaneously [21]. Prediction maps can provide more detailed information on how many and where attributes are exactly located. The pH changes of large Japanese sausages under different storage conditions were investigated by HSI via a distribution map developed by R statistics [21]. The results showed that the pH values of samples stored at 35 °C for 1, 3, and 5 days started to decrease in the surrounding area [19]. Compared to a conventional digital pH meter that can only identify the measuring spot, distribution maps generated by HSI can elaborate on how many different pH values exist in every spot in different test samples. The Cantonese sausage grade was classified by using multiple linear regression (MLR) and partial least squares regression (PLSR), and the best classification was achieved by using a successive projections algorithm coupled with MLR, with a predictive accuracy of 100% [22]. Shi et al. (2019) employed HSI to classify colonies from food fragments (sausage, bacon, and millet fragments) in an agar plate [23]. Kamrruzzaman et al. (2022) studied the moisture of red meat and corn using six different methods to select the wavelength in terms of model performance and demonstrated that competitive adaptive reweighted sampling (CARS) in tandem with PLSR is superior to PLSR models when developing models between the moisture and spectra of red meat and corn using a full spectral range [24]. Siripatrawan and Makino (2018) evaluated the changes in physicochemical, microbiological, and sensory attributes of packaged bratwurst (a type of sausage) during 20-day storage at 4 ± 1 °C [25]. Lightness, thiobarbituric acid, total viable counts, lactic acid bacteria, odor, and overall acceptability were found to be highly correlated with reflectance [25]. All these studies accentuate an unyielding interest in the application of HSI as an emerging method to detect different types of meat products.

Although previous studies have investigated the pH [26], color [27,28,29], and triphosphate content [30] of sausages by using HSI, scarce information has been obtained on evaluating the effects of extracts from waste orange peels on the pH of sausages after addition in a modified solution by using RSM and HSI combined with the PLSR algorithm. The novelty of the current study is:

(1) The simultaneous effects of five variables (different concentrations of soy lecithin, soy oil, lactic acid, orange extracts, and treatment times) on the pH of sausages during 15 days of storage were elucidated by RSM.
(2) The pH of sausages stuffed in modified casings after adding orange extracts was elaborated for the first time by HSI. The pH changes of each pixel in casings responding to different concentrations of orange extracts were clearly illustrated via prediction maps. The results can provide a better understanding of how pH reacts with orange extracts and provide useful information for future investigations.
(3) The relationship between pH and spectra from the surface of cylindrical sausages with modified casings was established for the first time by PLSR.

The purpose of this study is to evaluate the effects of five variables on the pH of sausages by using RSM. Following this, a quantitative model relating spectral data to the reference pH of sausage with this new type of casing is established. Consequently, wavelengths with high predictive power are chosen, and the distribution map of pH with differently treated casings is developed using image processing algorithms.

## 2. Materials and Methods

### 2.1. Soxhlet Extraction

The peels of Valencia sweet orange (Citrus sinensis) were first dried at 45 °C for 7 days. The dried orange peels were finely blade-milled into powder, and an average of 40.70 ± 0.52 g of orange powder was added to a cartridge sealed with cotton for Soxhlet extraction using 250 mL of 100% methanol. The crude extracts were separated from the solvent using a rotary vacuum evaporator. The crude extracts were placed in a flow hood overnight, allowing the solvent to be fully evaporated. Subsequently, the crude extracts were washed with distilled water of twice their weights, filtered, dried, and stored in a desiccator to finally obtain the dried precipitate extracts for further usage. The efficiencies of crude extract and precipitation were calculated based on the weight ratio of crude and precipitation with orange powder. A total of 35 repetitions were conducted to obtain enough orange extracts.

### 2.2. Experiment Design and Casing Modification

The simultaneous effects of five treatment variables (soy lecithin (X_1_), soy oil (X_2_), orange extracts (X_3_), lactic acid (X_4_), and treatment time (X_5_)) on pH were studied using RSM. The ranges of the independent variables were defined as follows: 2.12–4.20% for X_1_, 1.18–2.39% for X_2_, 0.12–0.40% for X_3_, 18–21 mL/kg NaCl for X_4_, and 60–90 min for X_5_. The experimental plan was designed by Minitab 21.1 software with an alpha (α) of 2 (Kozo Keikaku Engineering Inc., Tokyo, Japan). A total of 32 experiment matrices, including 6 replicates of the central point, were conducted in a random order to avoid erroneous conclusions due to extraneous sources of variability [1,17]. A quadratic polynomial equation was employed to establish the relationship between Y_pH_ and independent variables (X_a_; a = 1 − 5).

(1) $$Y{\mathrm{pH}}_{\;}=\beta +\sum_{a=1}\beta_{a}X_{a\;}+\sum_{a=1}\beta_{aa}X_{a}X_{a\;}+\sum_{a=1}\sum_{b=a+1}\beta_{ab}X_{a}X_{b}$$

where β, β_a_, β_aa_, and β_ab_ are the coefficients of constant, linear, quadratic, and interaction, respective. The F test was employed to estimate the significance of the regression parameters.

The surfactant solution was made by dissolving soy lecithin and soy oil with distilled water via magnetic agitation at 500 rpm and 60 °C. The orange extracts were added when the modified solution was cooled to 25 °C and mixed well with the magnetic agitation. A length of 30 cm natural hog casing (Pakumogu.com, Niigata Prefecture, Japan) section was desalted and placed in surfactant solution added with orange extracts with magnetic agitation at 500 rpm. The casings after treatment time were picked up and placed in the slush salt with lactic acid for another treatment time. The composition of the surfactant solution with orange extracts, slush salt with lactic acid, and treatment time are shown in Table 1.

### 2.3. Sausage Production

Sausage batter was made of lean pork (4020 g), fat (1720 g), Chinese white wine (718.6 g; ethanol content: 52%, *v*/*v*), salt (200 g), sugar (120 g), black pepper (73.9 g), coriander powder (37.6 g), spicy pepper (42.4 g), turmeric powder (16.8 g), and garam masala powder (17.5 g). The meat and fat were sterilely cut into small pieces and mixed well with spices and wine. The mixed batter was filled into the modified casings and desalted untreated natural hog casings (as a control) by using a stuffing machine (STX-4000-TB2-PD-BL, Electric Meat Grinder & Sausage Stuffer, STX International, Lincoln, NE, USA). The sausage sections after stuffing were immediately dried in a sterilized oven at 45 °C for 24 h and aged at 20 °C for another 48 h. Subsequently, the sausage sections were vacuum packaged and stored in a fridge (4 °C) for 16 days for the following post-analysis.

### 2.4. Hyperspectral Imaging System

A laboratory visible near-infrared HSI (NH-4-KIT, EBA Japan, Tokyo, Japan) with a spectra range of 350 nm–1100 nm was employed for push-broom line-scanning of the sausage surface. A 10-bit charged coupled device camera was used, and the flame rate was 100 fps with an exposure time of 12.47 ms. The total contiguous spectral bands were 151 with 5 nm intervals. The halogen lamp light source was fixed on three sides of the camera, and the white sheet was used to render the light source to have an even distribution. The HSI system was calibrated by a dark reference (with 0% reflectance) by completely covering the camera lens with its opaque cap and calibrated by a white reference with 100% reflectance before measuring. Imaging acquisition was conducted using a reflectance mode of HSI with a dark room temperature of 20 °C. The corrected images reflectance (R_ci_) was expressed as the following equation using the software of the imaging system

(2) $$R_{\mathrm{ci}}=\frac{R_{r}-R_{d}}{R_{w}-R_{d}}$$

where R_r_, R_d_, and R_w_ are the reflectance images of raw, dark, and white, respectively. The absorbance profiles were transformed using the following equations:

A = −log R(3)

A black background (low reflectance surface) was used to achieve a good contrast between the sample and the background. Control software (HSAnalyzer, version 1.2, EBA Japan, Tokyo, Japan), supported by a computer, was used to analyze and process the hyperspectral images. The hypercubes were segmented by applying a threshold criterion. In this sense, all pixels having a reflectance higher than 0.075 at band 70 (695 nm) were considered as sausage. Moreover, several spectra from each sample, each sausage was digitally divided into five regions of equal area. The average spectrum of each segment was extracted and merged in a table that also contained the reference pH value and the sample code. The segmentation, the extraction of the spectra as well as the storage of data were developed by an algorithm coded under MATLAB (MathWorks Inc., Natick, MA, USA). This table with both spectra and pH reference values was used to develop the calibration models.

### 2.5. pH Analysis

Ten grams of sausage slices was homogenized with 90 mL of distilled water for 1 min and measured using a digital pH meter. The method was based on Zdolec et al. (2008) [31].

### 2.6. Spectral Pretreatment and Model Development

In order to enhance the model performance, the pretreatments of spectra, such as multiplicative scatter correction (MSC), standard normal variate (SNV), normalization, the first and the second derivatives, were conducted before multivariate analysis. Partial least square regression (PLSR), as one of the important regression algorithms, was employed to connect the pH parameter and the spectra of the sausages stuffed with different treated modified casings. Two-thirds of the samples were used for the calibration group, while the left one-third was used for the prediction group.

The predictive ability was estimated by the determination coefficient in calibration (R_c_^2^), prediction (R_p_^2^), cross-validation (R_cv_^2^), the root mean square errors of calibration (RMSEC), prediction (RMSEP), and cross-validation (RMSECV). These parameters were determined as follows:

(4) $$R^{2}=1-\frac{\sum^{}{\left(y_{i\;}-y_{predicted}\right)}^{2}}{\sum^{}{\left(y_{i\;}-y_{mean}\right)}^{2}}$$

(5) $$\mathrm{RMSE}=\sqrt{\sum_{i=1}^{n}\frac{{\left(y_{i\;}-y_{predicted}\right)}^{2}}{n}}$$

where *y_i_* is the measured pH of the *i*th sample, and *n* is the number of samples. A good PLSR model is commonly believed to have a high R^2^ with a low RMSE with a small absolute difference between RMSEC and RMSECV.

### 2.7. Feature Wavelengths Selection

The feather wavelengths (FWs) for pH were selected based on the weighted regression coefficient from the developed calibration model, to simplify the full model and reduce noise and redundancy information and so enhance the model accuracy. Wavelengths that contained large regression coefficients (irrespective of the sign) were selected as the FWs, and a new simplified model was created using those FWs. If the predictive performance of the new model is comparable to that with full spectra, it could be of practical use to design a simple cost-effective multispectral system and applicable to commercialization.

### 2.8. Visualization of pH Distribution

**Figure 1:**
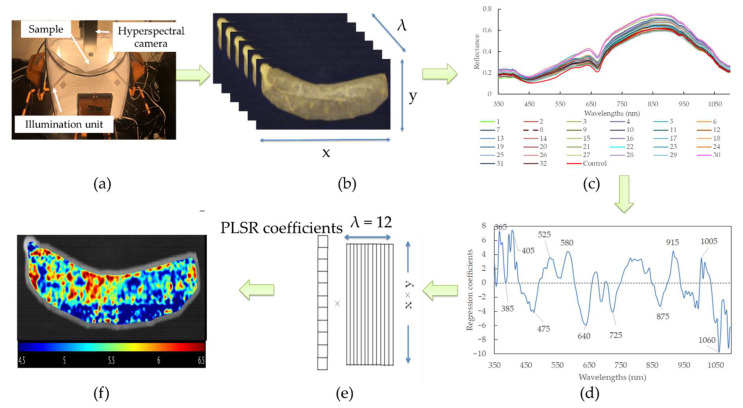
Flowchart of the main steps to design a hyperspectral imaging system for real-time monitoring of pH in sausages with different modified casings. (**a**) Imaging acquisition; (**b**) hyperspectral image segmentation; (**c**) spectral extraction; (**d**) feature wavelengths selection; (**e**) unfolded image to 2D and multiply the regression coefficients of PLSR model using feature wavelengths; (**f**) prediction map establishment.

The pH prediction maps for sausages with different modified casings were produced according to calibration models with FWs. As HSI possesses a 3D matrix that contains a large amount of spatial and spectral information, it is required to unfold into a 2D matrix at the FWs. The purpose of this is to render each row standing for the spectrum of a pixel, and the columns represented the selected feather wavelengths. The matrix was then multiplied by the corresponding regression coefficient. In this way, the vector could be refolded back to generate a 2D color image, and consequently, the color heterogeneity of the sausages with a linear color scale (i.e., distribution map) could be shown. All these procedures were computed with MATLAB software (R2017b; MathWorks Inc., Natick, MA, USA). The graphical scheme of the main steps of building the prediction map is illustrated in Figure 1.

## 3. Results and Discussion

### 3.1. Spectral Characteristics and Simultaneous Effects on pH Analyzed by RSM

#### 3.1.1. Spectra Overview

**Figure 2:**
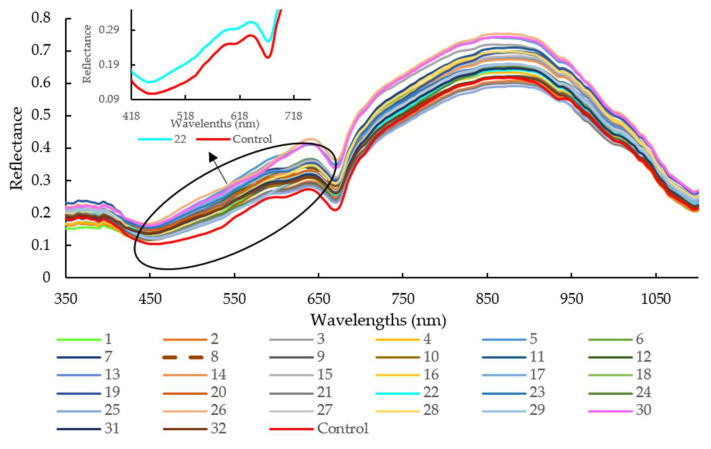
Spectra of all raw samples with different modified casing treatments in the spectral range of 350 to 1100 nm. Note: legend number means different treatments

As illustrated in Figure 2, the average reflectance of sausages stuffed in the modified casing with Treatment 30 was higher than that with Treatment 25 and the control group, especially for the wavelengths range of 645 nm to 670 nm and 865 nm to 890 nm. The pH values for Treatments 30, 25, and control were 4.44, 6.32, and 6.24, respectively. It is well known that the NIR hyperspectral system recorded the different absorbance patterns according to the interior structural changes or intermolecular forces changes in the meat with different pH levels [32]. It was also reported that oxymyoglobin (MbO_2_) has a large absorption coefficient at 544 nm and 582 nm, while oxyhemoglobin (HbO_2_) possesses three absorption bands at 418 nm, 543 nm, and 574 nm, representing soret, α, and β bands [33]. The absorption bands at 780 and 980 nm were related to moisture absorption, corresponding to the third and second overtones of O-H stretching, respectively [34].

#### 3.1.2. Simultaneous Effects on pH Analyzed by RSM

**Figure 3:**
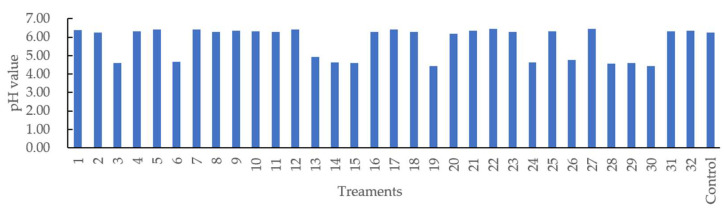
pH value of the sausages stuffed in modified casings with different treatments.

Figure 3 shows that the pH value varied from 4.44 (Treatments 19 and 30) to 6.46 (Treatment 22). Different casing treatments may have affected pH evaluation during the 16 days of storage under 4 °C. The R^2^ value of the regression model developed for pH was 67.13% with a nonsignificant lack of fit (*p* > 0.05). Only soy lecithin and orange extracts (X_1_ × X_3_) were observed to have interactively affected the pH of sausage at a 5% significance level (Table 2). The predicted quadratic polynomial regression equation for pH as a function of soy lecithin (X_1_), soy oil (X_2_), orange extracts (X_3_), lactic acid (X_4_), and treatment time (X_5_) in the uncoded units are shown as follows:

(6) $$Y_{\mathrm{pH}}=59.0000+1.5200\;X_{1}-9.0700\;X_{2}-8.4000\;X_{3}-3.8400\;X_{4}-0.2520\;X_{5}-0.0520\;X_{1}^{2}+0.4260\;X_{2}^{2}+7.8200\;X_{3}^{2}+0.0635\;X_{4}^{2}+0.0007\;X_{5}^{2}+0.0110\;X_{1}X_{2}-3.2700\;X_{1}X_{3}-0.0160\;X_{1}X_{4}-0.0035\;X_{1}X_{5}+2.0700\;X_{2}X3_{\;}+0.2720\;X_{2}X_{4}+0.0216\;X_{2}X_{5}+1.0000\;X_{3}X_{4}-0.1109\;X_{3}X_{5}+0.0086\;X_{4}X_{5}$$

**Figure 4:**
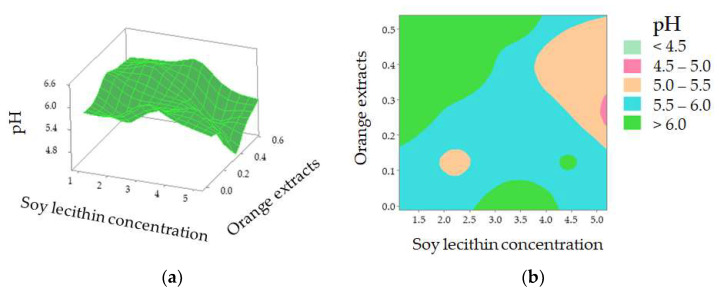
pH value of the sausages stuffed in modified casings as affected by soy lecithin concentration and addition of orange extracts (**a**); 2D contour plot (**b**).

The value of the corresponding coefficient for orange extracts (X_3_, −8.4000) indicates that it exerts an important impact on casing modification and so influenced sausage pH during the 16-day storage. Further investigation shows that pH decreased when casings were treated with a higher concentration of soy lecithin with orange extracts at approximately 0.26% [Figure 4a]. According to the 2D contour plot [Figure 4b], the pH value is over 6.0 when a lower soy lecithin concentration was combined with higher orange extracts. The increased pH may be explained by the nitrogen compounds generated by proteolysis [35]. Yao et al. reported that protein enzymes, produced by microorganisms, led to the decomposition of meat protein, generating ammonia, biogenic amine, and skatole [33]. The contents of MbO_2_ and HbO_2_ were decreased with the decomposition of protein, which indirectly affected the change of spectra at 540 and 582 nm (related to MbO_2_), and 418, 543, and 574 nm (related to HbO_2_) in comparison with the control (pH = 6.24) and sample with Treatment 22 (pH = 6.46) (Figure 2). A lower soy lecithin level was reported to render the casings to be more porous [1]. Orange extracts were reported to possess certain contents of hesperidin [9,17,36,37,38], which has a potent antioxidant effect and is an anti-microorganism.

### 3.2. Calibration Models with Full Wavelengths

The PLSR model was employed to predict the pH values of the sausages using different modified casings from both reflectance and absorbance spectral data over the full spectral range of 350–1100 nm. Using the reflectance spectra, the R_p_^2^ value of data pretreated by normalization shows some improvement to 0.6855 when compared with the raw data of 0.6485, with a small decrease in RMSEP (Table 3). It is reported that normalization can improve the spectral features, making the spectra have an equal area under the curve to allow for easy comparison of spectral features in the same plot [39]. Proper normalization of hyperspectral spectra data enables a better signal-to-noise ratio, compared to the conventional spectrophotometer method [40] A-PLSR model derived from the spectra preprocessed by the first derivative presented the highest R_c_^2^ of 0.7300, the lowest RMSEC of 0.4283%, and R_p_^2^ of 0.6789 and RMSEP of 0.4501% (Table 3), with a low absolute difference between RMSEC and RMSECV, compared with raw and other pretreatments. The function of first derivatives is to remove background noise and baseline drift, as well as to improve small spectral features [41]. Regarding a similar study, the R_p_^2^ values for predicting pH in salted meat [42], freeze–thawed pork [43], fresh pork [44], and large Japanese sausages [19] were 0.797, 0.845, 0.55, and 0.882, respectively. The comparable low R_p_^2^ in the current study may be due to the factor where the pH value was only concentrated either near 4.66 or 6.32 (Figure 3), leading to a narrow pH distribution for analysis.

### 3.3. Calibration Models with Selected Feature Wavelengths

**Figure 5:**
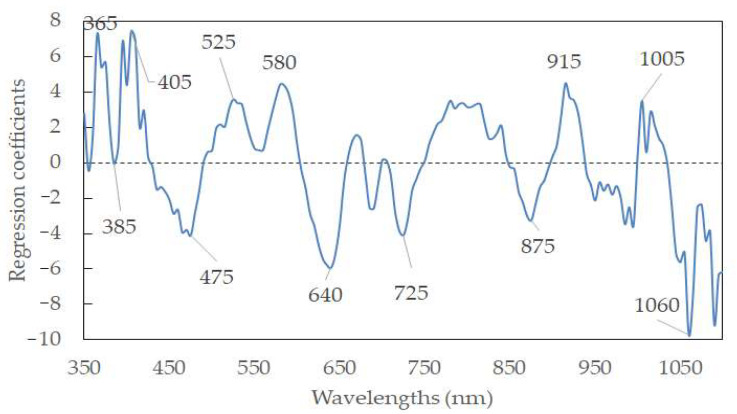
Feature wavelength selection from the PLSR model.

Hyperspectral data display a high degree of interband correction with high dimensions. It is thus meaningful to select feature wavelengths to represent the full wavelengths. In this way, the model can be simplified, and data redundancy can be eliminated. Furthermore, the algorithms’ efficacies can be enhanced, accelerating the classification of sausages based on the pH for industrial machine-vision systems. According to the weighted regression coefficients from the PLSR model (Figure 5), the feature wavelengths were selected based on the peaks and valleys (i.e., indicating the important information and large contribution to the productivity of the model) in the wavelengths. To this end, twelve wavelengths (365, 385, 405, 475, 525, 580, 640, 725, 875, 915, 1005, and 1060 nm) were selected as the feature wavelengths. Table 4 elaborates on the predictive ability of the simplified model, with an R_p_^2^ of 0.6896 and an RMSEP of 0.4426%, using data pretreated by normalization. A new PLSR with those feature wavelengths was established as follows:

Y’_pH_ = 5.335 + 7.243λ_365_ − 0.060λ_385_ + 7.461λ_405_ − 4.098λ_475_ + 3.594λ_525_ + 4.443λ_580_ − 5.920λ_640_ − 4.083λ_725_ − 3.246λ_875_ + 4.495λ_915_ + 3.520λ_1005_ − 9.730λ_1060_ (7)

where λ_xnm_ is the reflectance spectra at the wavelength of x nm, and Y’_pH_ is the predicted pH value. Generally, the R^2^ values of prediction in the reduced model were comparable to that of the model using full wavelengths, and approximately 92% of wavelengths were reduced from the full wavelengths. There were even some cases where the prediction accuracy of the model using the feature wavelengths was higher than that using the full wavelengths (e.g., R_p_ = 0.6835 for A-1st Derivative-PLSR using feature wavelength vs. R_p_ = 0.6789 for A-1st Derivative-PLSR using full wavelength). This may be elucidated by removing the noise and pixel outliers, resulting in the improvement of robustness and reliability of the PLSR model [44,45,46,47]. It was mentioned that the R^2^ value between 0.66 and 0.81 is allowed for approximate prediction, an R^2^ value between 0.82 and 0.90 is regarded as a good prediction, while an R^2^ higher than 0.91 is demonstrated to be an excellent prediction [48]. The selected feature wavelengths were applied to creating prediction maps in the following step.

### 3.4. Visual Representation of Sausage pH Distribution

**Figure 6:**
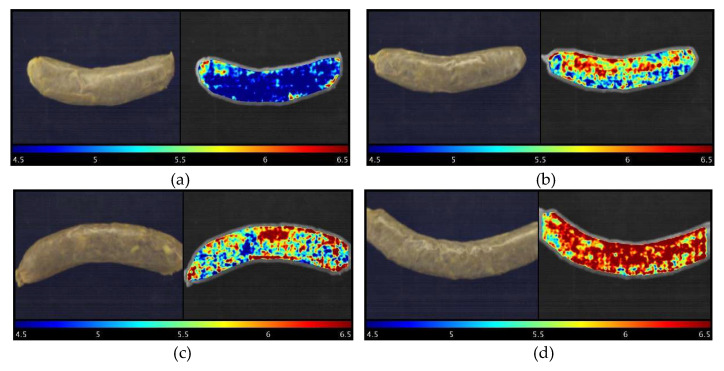
RGB images (left) and visualization of pH prediction map (right) for sausages stuffed in different treated modified casings stored on Day 16. Note: (**a**): Treatment 30 sample, pH = 4.44; (**b**): Treatment 20 sample, pH = 6.17; (**c**): Control sample, pH = 6.24; and (**d**): Treatment 22 sample, pH = 6.46.

As the main advantage of hyperspectral imaging technology, a prediction map (or distribution map) can illustrate where and how many different reference parameters (pH in the current study) are located from spot to spot in different detected samples. Figure 6 elaborates how pH changes in response to the different treated modified casings used for the sausages stored on Day 16. Four samples, the sample with the highest pH value (treatment 22, pH = 6.46), middle pH (Treatment 20, pH = 6.17), lowest pH (Treatment 30, pH = 4.44), and control group, were selected for representation. It is difficult to distinguish the differences in the RGB images with the naked eyes (Figure 6). Nevertheless, the prediction maps clearly show how much different pH values were located from pixel to pixel, which is superior to the conventional pH measurement, which is time-consuming and difficult to obtain the data like this. It is meaningful to monitor and understand the spatial change, which could provide useful information on how intrinsic properties such as the composition and biochemical attributes of samples react to different modified casings. To this point, it could serve as a good reference before those types of modified casings are commercially utilized.

## 4. Conclusions

The present study investigated the feasibility of employing HSI for rapidly and nondestructively predicting pH values in sausages stuffed in modified casings added with orange extracts from waste orange peels. RSM was used to explain the relationship between different modified factors and sausage pH. The R^2^ value of the polynomial regression model was 67.13% with a nonsignificant lack of fit (*p* > 0.05). When using full wavelengths, the A-PLSR model derived from the spectra preprocessed by the first derivative presented the highest R_c_^2^ of 0.7300, while R-PLSR derived from normalization achieved the highest R_p_^2^ of 0.6855. Twelve feature wavelengths (365, 385, 405, 475, 525, 580, 640, 725, 875, 915, 1005, and 1060 nm) were selected to develop a simplified model. This reduced model achieved a comparable R^2^ value in comparison with that using full wavelengths, which means it can be applied to a simple, cost-effective, multispectral system establishment or for online industrial applications. For the first time, the prediction map developed in this study clearly displayed sausage pH evolution according to different modified casings.

## Figures and Tables

**Table 1:** Casing modification treatment combinations.

Treatments	Surfactant Solution with Orange Extracts				Slush Slat with Lactic Acid	
	Soy Lecithin Concentration (X_1_, %, *w*/*w*)	Soy Oil Concentration (X_2_,%, *w*/*w*)	Orange Extracts (X_3_, %, *w*/*w*)	Treatment Time (X_5_, min)	Lactic Acid (ml/kg NaCl, X_4_)	Treatment Time (X_5_, min)
1	3.16 (C, 0)	1.78 (C, 0)	0.26 (C, 0)	75 (C, 0)	19.50 (C, 0)	75 (C, 0)
2	3.16 (C, 0)	1.78 (C, 0)	0.26 (C, 0)	75 (C, 0)	22.50 (A, +α)	75 (C, 0)
3	4.20 (F, +1)	1.18 (F,−1)	0.40 (F, +1)	90 (F, +1)	18.00 (F,−1)	90 (F, +1)
4	3.16 (C, 0)	1.78 (C, 0)	0.26 (C, 0)	75 (C, 0)	16.50 (A, -α)	75 (C, 0)
5	2.11 (F, −1)	2.38 (F, +1)	0.12 (F, −1)	90 (F, +1)	21.00 (F, +1)	90 (F, +1)
6	3.16 (C, 0)	1.78 (C, 0)	0.26 (C, 0)	75 (C, 0)	19.50 (C, 0)	75 (C, 0)
7	2.11 (F, −1)	2.38 (F, +1)	0.40 (F, +1)	60 (F, −1)	21.00 (F, +1)	60 (F, −1)
8	1.07 (A, -α)	1.78 (C, 0)	0.26 (C, 0)	75 (C, 0)	19.50 (C, 0)	75 (C, 0)
9	3.16 (C, 0)	1.78 (C, 0)	0.26 (C, 0)	105 (A, +α)	19.50 (C, 0)	105 (A, +α)
10	3.16 (C, 0)	1.78 (C, 0)	0.26 (C, 0)	45 (A, -α)	19.50 (C, 0)	45(A, -α)
11	3.16 (C, 0)	1.78 (C, 0)	0.26 (C, 0)	75 (C, 0)	19.50 (C, 0)	75 (C, 0)
12	2.11 (F, −1)	1.18 (F, −1)	0.40 (F, +1)	90 (F, +1)	21.00 (F, +1)	90 (F, +1)
13	4.20 (F, +1)	2.38 (F, +1)	0.12 (F, −1)	60 (F, −1)	21.00 (F, +1)	60 (F, −1)
14	4.20 (F, +1)	2.38 (F, +1)	0.40 (F, +1)	60 (F, −1)	18.00 (F, −1)	60 (F, −1)
15	4.20 (F, +1)	1.18 (F, −1)	0.40 (F, +1)	60 (F, −1)	21.00 (F, +1)	60 (F, −1)
16	2.11 (F, −1)	1.18 (F, −1)	0.12 (F, −1)	90 (F, +1)	18.00 (F, −1)	90 (F, +1)
17	3.16 (C, 0)	2.93(A, +α)	0.26 (C, 0)	75 (C, 0)	19.50 (C, 0)	75 (C, 0)
18	4.20 (F, +1)	1.18 (F, −1)	0.12 (F, −1)	90 (F, +1)	21.00 (F, +1)	90 (F, +1)
19	2.11 (F, −1)	1.18 (F, −1)	0.12 (F, −1)	60 (F, −1)	21.00 (F, +1)	60 (F, −1)
20	4.20 (F, +1)	2.38 (F, +1)	0.40 (F, +1)	90 (F, +1)	21.00 (F, +1)	90 (F, +1)
21	2.11(F, −1)	1.18 (F, −1)	0.40 (F, +1)	60 (F, −1)	18.00 (F, −1)	60 (F, −1)
22	4.20(F, +1)	1.18 (F, −1)	0.12 (F, −1)	60 (F, −1)	18.00 (F, −1)	60 (F, −1)
23	3.16 (C, 0)	1.78 (C, 0)	0.53(A, +α)	75 (C, 0)	19.50 (C, 0)	75 (C, 0)
24	5.16 (A, +α)	1.78 (C, 0)	0.26 (C, 0)	75 (C, 0)	19.50 (C, 0)	75 (C, 0)
25	2.11 (F, −1)	2.38 (F, +1)	0.40 (F, +1)	90 (F, +1)	18.00 (F, −1)	90 (F, +1)
26	3.16(C, 0)	1.78(C, 0)	0.26 (C, 0)	75 (C, 0)	19.50 (C, 0)	75 (C, 0)
27	4.20 (F, +1)	2.38 (F, +1)	0.12 (F, −1)	90 (F, +1)	18.00 (F, −1)	90 (F, +1)
28	3.16 (C, 0)	1.78(C, 0)	0.26 (C, 0)	75 (C, 0)	19.50 (C, 0)	75 (C, 0)
29	3.16 (C, 0)	1.78(C, 0)	0.26 (C, 0)	75 (C, 0)	19.50 (C, 0)	75 (C, 0)
30	2.11 (F, −1)	2.38 (F, +1)	0.12 (F, −1)	60 (F, −1)	18.00 (F, −1)	60(F, −1)
31	3.16(C, 0)	1.78 (C, 0)	0.00 (A, -α)	75 (C, 0)	19.50 (C, 0)	75 (C, 0)
32	3.20(C, 0)	0.60 (A, -α)	0.26 (C, 0)	75 (C, 0)	19.50 (C, 0)	75 (C, 0)

Note: A: axial, C: center, F: factorial; the numbers α −1, 0, 1, and +α in the brackets mean coded variable level.

**Table 2:** Regression coefficients and analysis of variance of the regression models for pH.

Analysis of Variance			
Source	Df	Adj SS	F-Value
Model	20	14.45	1.12
Linear	5	3.54	1.10
X_1_	1	1.64	2.56
X_2_	1	0.01	0.01
X_3_	1	0.00	0.01
X_4_	1	0.00	0
X_5_	1	1.88	2.93
Square	5	2.55	0.79
X_1_ × X_1_	1	0.12	0.19
X_2_ ×X_2_	1	0.81	1.27
X_3_ ×X_3_	1	0.64	1.00
X_4_ ×X_4_	1	0.60	0.93
X_5_ × X_5_	1	0.71	1.10
2-Way Interaction	10	8.37	1.30
X_1_ × X_2_	1	0.00	0.00
X_1_ × X_3_	1	3.98	6.19 *
X_1_ × X_4_	1	0.01	0.02
X_1_ × X_5_	1	0.06	0.09
X_2_ × X_3_	1	0.50	0.78
X_2_ × X_4_	1	1.04	1.62
X_2_ × X_5_	1	0.66	1.02
X_3_ × X_4_	1	0.68	1.06
X_3_ × X_5_	1	0.84	1.30
X_4_ × X_5_	1	0.60	0.93
Error	11	7.07	
Lack of Fit	6	3.30	0.73
Pure Error	5	3.78	
Total	31	21.52	

Note: * significant at *p* < 0.05, without any mark means not significant. Adj SS: adjust sum of squares, Df: degree of freedom.

**Table 3:** Statistical parameters of PLSR with raw and preprocessing spectra based on full wavelengths.

	Treatments	Calibration Group (*n* = 110)		Prediction Group (*n* = 55)		Cross-Validation	
Reflectance (R)		**R_c_^2^**	**RMSEC (%)**	**R_p_^2^**	**RMSEP (%)**	**R_cv_^2^**	**RMSECV (%)**
	Raw	0.6849	0.4628	0.6485	0.4709	0.7496	0.4063
	1st Derivative	0.5342	0.5626	0.6401	0.4766	0.8756	0.2864
	2nd Derivative	0.6291	0.502	0.3843	0.6233	0.7431	0.4115
	MSC	0.5569	0.5488	0.6416	0.4756	0.6524	0.4787
	SNV	0.5570	0.5487	0.6416	0.4756	0.6528	0.4785
	Normalization	0.6770	0.4685	0.6855	0.4455	0.7194	0.4301
	Normalization + 1st Derivative	0.6813	0.4654	0.6503	0.4698	0.7152	0.4333
	1st Derivative + Normalization	0.5692	0.5411	0.5524	0.5314	0.6197	0.5007
	Normalization + 2nd Derivative	0.7282	0.4298	0.4091	0.6106	0.7534	0.4032
	2nd Derivative + Normalization	0.3417	0.6688	0.1309	0.7406	0.3447	0.6573
Absorbance (A)	Raw	0.7025	0.4496	0.6626	0.4614	0.7305	0.4216
	1st Derivative	0.7300	0.4283	0.6789	0.4501	0.7798	0.3810
	2nd Derivative	0.5257	0.5677	0.3710	0.63	0.6544	0.4733
	MSC	0.5479	0.5543	0.5677	0.5223	0.6384	0.4883
	SNV	0.5476	0.5545	0.5680	0.5221	0.6384	0.4883
	Normalization	0.5021	0.5817	0.6015	0.5015	0.5927	0.5182
	Normalization + 1st Derivative	0.5324	0.5637	0.6560	0.4659	0.5706	0.5321
	1st Derivative + Normalization	0.4812	0.5938	0.4831	0.5711	0.6123	0.5056
	Normalization + 2nd Derivative	0.6149	0.5116	0.2514	0.6873	0.6240	0.4979
	2nd Derivative + Normalization	0.0179	0.8170	0.0217	0.7857	0.0183	0.8045

Note: MSC: multiplicative scatter correction; SNV: standard normal variate; RMSEC: root mean square error of calibration; RMSEP: root mean square error of prediction; RMSECV: root mean square error of cross-validation.

**Table 4:** Statistical parameters of PLSR with raw and preprocessing spectra based on selected feature wavelengths.

	Treatments	Calibration Group (*n* = 110)		Prediction Group (*n* = 55)		Cross-Validation	
Reflectance		**R_c_^2^**	**RMSEC (%)**	**R_p_^2^**	**RMSEP (%)**	**R_cv_^2^**	**RMSECV (%)**
	Raw	0.6876	0.4608	0.6648	0.4599	0.6844	0.4561
	1st Derivative	0.5881	0.5291	0.6257	0.486	0.6373	0.4890
	2nd Derivative	0.5560	0.5493	0.6640	0.4604	0.6001	0.5135
	MSC	0.5370	0.5609	0.6530	0.4680	0.6319	0.4926
	SNV	0.5369	0.5610	0.6532	0.4678	0.6229	0.4986
	Normalization	0.6860	0.4619	0.6896	0.4426	0.6954	0.4482
	Normalization + 1st Derivative	0.6695	0.4739	0.6613	0.4623	0.6705	0.4661
	1st Derivative + Normalization	0.6124	0.5132	0.6189	0.4904	0.6021	0.5122
	Normalization + 2nd Derivative	0.6603	0.4805	0.6688	0.4571	0.6730	0.4643
	2nd Derivative + Normalization	0.5427	0.5574	0.6393	0.4771	0.5956	0.5164
Absorbance	Raw	0.6996	0.4518	0.6706	0.4559	0.7050	0.4410
	1st Derivative	0.6553	0.4840	0.6835	0.4469	0.7026	0.4428
	2nd Derivative	0.6608	0.4801	0.6653	0.4596	0.6654	0.4697
	MSC	0.5564	0.549	0.5824	0.5133	0.5709	0.5319
	SNV	0.5209	0.5706	0.6267	0.4854	0.5912	0.5191
	Normalization	0.5719	0.5394	0.5315	0.5437	0.5956	0.5163
	Normalization + 1st Derivative	0.5331	0.5633	0.5782	0.5159	0.5616	0.5376
	1st Derivative + Normalization	0.5517	0.5519	0.5307	0.5442	0.5413	0.5499
	Normalization + 2nd Derivative	0.5899	0.5279	0.5822	0.5135	0.5973	0.5153
	2nd Derivative + Normalization	0.5892	0.5284	0.5728	0.5192	0.5977	0.515

Note: MSC: multiplicative scatter correction; SNV: standard normal variate; RMSEC: root mean square error of calibration; RMSEP: root mean square error of prediction; RMSECV: root mean square error of cross-validation.

## Data Availability

Data are contained within this article.
